# Regular consumption of Lactobacillus reuteri‐containing lozenges reduces pregnancy gingivitis: an RCT

**DOI:** 10.1111/jcpe.12606

**Published:** 2016-08-12

**Authors:** Ulrich Schlagenhauf, Lena Jakob, Martin Eigenthaler, Sabine Segerer, Yvonne Jockel‐Schneider, Monika Rehn

**Affiliations:** ^1^ Department of Periodontology University Hospital Wuerzburg Wuerzburg Germany; ^2^ Department of Orthodontics University Hospital Wuerzburg Wuerzburg Germany; ^3^ Department of Obstetrics and Gynaecology University Hospital Wuerzburg Wuerzburg Germany

**Keywords:** *L. reuteri*, plaque, pregnancy, pregnancy gingivitis

## Abstract

**Aim:**

This randomized controlled trial assessed the impact of *Lactobacillus reuteri* on pregnancy gingivitis in healthy women.

**Materials and Methods:**

Forty‐five healthy women (24 test/21 placebo) with pregnancy gingivitis in the third trimester of pregnancy were enrolled. At baseline Gingival Index (GI) and Plaque Index (PlI) were assessed at the Ramfjord teeth and venous blood taken for TNF‐*α* analysis. Subsequently participants were randomly provided with lozenges to be consumed 2 × daily until birth (approx. 7 weeks) containing ≥10^8^ CFU *L. reuteri* ATCC PTA 5289 and ≥10^8^ CFU *L. reuteri* DSM 17938 (test) or being devoid of *L. reuteri* (placebo). Within 2 days after birth recording of GI, PlI and blood sampling were repeated.

**Results:**

At baseline, mean GI and mean PlI did not differ significantly between both groups. In the test group mean TNF‐*α* serum level was significantly (*p* < 0.02) lower than in the placebo group. At reevaluation, mean GI and mean PlI of the test group were both significantly (*p* < 0.0001) lower than in the placebo group. Mean TNF‐*α* serum level did no longer differ significantly between the groups.

**Conclusions:**

The consumption of *L. reuteri* lozenges may be a useful adjunct in the control of pregnancy gingivitis.

The development of gingivitis is a very frequent corollary of pregnancy with a reported prevalence of 35–100% (Wu et al. 2015). In clinical practice it is usually controlled by professional tooth cleaning and the establishment of adequate oral hygiene measures (Kaur et al. 2014). Nevertheless already the ground breaking experiments by Löe and Silness (Loe & Silness 1963, Silness & Loe 1964) suggested, that pregnancy gingivitis is not identical to common plaque‐associated gingivitis due to a significant shift in the correlation between observed bacterial plaque mass and the ensuing extent of gingival inflammation before and after pregnancy. Subsequent evidence from in vitro studies and clinical trials confirmed and further corroborated the validity of this distinction (Raber‐Durlacher et al. 1993, 1994, Niederman 2013, Wu et al. 2015). The putative etiological explanation has been ascribed to the manifestation of various histological, serological and immunological changes within the gingival tissues during pregnancy. These result in a significant increase in the crevicular fluid flow rate and a concomitant rise at the concentration of proinflammatory cytokines as well as pregnancy‐associated sex hormones, favouring the overgrowth of gingivitis‐inducing microorganisms (Kornman & Loesche 1980, Fteita et al. 2015, Lima et al. 2015, Wu et al. 2015). Kornman and Loesche reported that in the second trimester of pregnancy the strongly increased recovery of *Prevotella intermedia* from inflamed gingival sites was correlated with increased plasma levels of estrogen and progesterone both providing a substitute for menadione an essential *P. intermedia* growth factor. Nevertheless a clear distinction between pregnancy‐associated gingivitis and general gingivitis based on the composition of the oral microbiome has not been possible so far (Kumar 2013).

A steadily increasing number of studies in recent years on the interplay between microorganisms and the human host at mucosal barriers of the intestine have substantially broadend the insights on the mechanisms involved. Unlike traditional views mostly focusing on virulence mechanisms of microbial pathogens and their impact on host response, there is now sufficient evidence that the functionality of the mucosal immune system is also intimately influenced by the commensal microbiota (Sansonetti 2011). According to this concept disease and harmful inflammatory responses may not only originate from the proinflammatory stimuli of microbial pathogens but may also to a comparable extent be attributable to an eventual lack of protective commensal microorganisms (Mazmanian et al. 2008). Under physiological conditions these dominate the mucosal interfaces and are actively inhibiting colonization by invading species. Furthermore cell wall antigens and metabolites of the commensal microbiota significantly shape the extent and direction of the mucosal immune response by influencing amongst others the cytokine profiles as well as the distribution of regulatory T‐cells and various T‐helper cell subtypes in the host (Alexander et al. 2014). Certain strains of *Lactobacillus reuteri*, commonly found in the gut and oral microbiota of healthy humans and other mammals have been identified as putative microorganisms, significantly modulating the status of intestinal and oral health. In vitro experiments assessing the metabolic properties of *L. reuteri*, revealed the synthesis of reutericyclin and reuterin, both broad spectrum antibiotics with a significant inhibitory effect on a wide array of bacterial species (Talarico et al. 1988, Ganzle et al. 2000, Baca‐Castanon et al. 2015). Data from a steadily growing number of clinical studies also demonstrated that *L. reuteri* exposure may induce a variety of beneficial host responses as diverse as reduced rates of allergies in infants, reduced caries activity in children and improved insulin secretion in healthy adults (Forsberg et al. 2013, Stensson et al. 2014, Simon et al. 2015). Furthermore in a controlled animal trial the addition of *L.reuteri* to the drinking water of mice led to a significant upregulation of the neuropeptide oxytocin, which cut the healing time of standarized skin wounds in half (Poutahidis et al. 2013). In controlled clinical trials finally, evaluating the regular consumption of *L. reuteri‐*containing food supplements in patients suffering from chronic gingivitis or periodontitis, a significant reduction in gingival bleeding and crevicular TNF‐*α* levels (Twetman et al. 2009) as well as significantly improved periodontal healing after scaling and root planning (Teughels et al. 2013, Tekce et al. 2015) was observed. This evidence however is still inconclusive as some other controlled clinical trials failed to demonstrate a significant effect of *L. reuteri* on clinical parameters of periodontal health (Iniesta et al. 2012, Hallstrom et al. 2013). Compared to plaque‐associated chronic gingivitis pregnancy‐associated gingivitis is characterized by a particularly complex interplay between hormonal changes, fluctuations in proinflammatory cytokines and the resulting shift of the oral microbiome. The administration of a probiotic *L. reuteri* food supplement in pregnancy gingivitis may thus have the potential for a beneficial, anti‐inflammatory interference with the resident microbiome as well as the host response independent of the establishment of efficacious oral hygiene. To our best knowledge an interventional trial focusing on the control of pregnancy‐associated gingivitis by the consumption of *L. reuteri* has not been published before.

## Aim

The purpose of this study was to assess the influence of the regular consumption of probiotic *L. reuteri*‐containing lozenges on the manifestation of pregnancy gingivitis in a cohort of healthy women during the third trimester of pregnancy.

## Material and Methods

### Lozenges

The lozenges (test, placebo) were manufactured and provided by BioGaia AB, Lund, Sweden. They were identical to the commercially available Prodentis lozenges made by BioGaia AB and consisted of isomalt (filler), hydrogenated palm oil, peppermint flavour, menthol flavour, peppermint oil and sucralose. Test lozenges furthermore contained ≥10^8^ colony forming units of *Lactobacillus reuteri* DSM 17938 and ≥10^8^ colony forming units of *Lactobacillus reuteri* ATCC PTA 5289, while placebo lozenges were void of *L. reuteri* strains. Test and placebo lozenges were stored in identical neutral bottles containing 28 lozenges each, with a label displaying a unique CRF subject number and the instruction to consume the lozenges 2× daily by slowly melting them in the mouth.

### Study design

The study was designed as a prospective, parallel group, two arm, double‐blind, placebo‐controlled randomized clinical trial. The study protocol was prepared in accordance with the declaration of Helsinki and met the criteria of good clinical practice; it was approved by the ethics committee of the University of Wuerzburg (file # 239/09). All subjects participating had signed a written informed consent prior to their inclusion.

### Study population

Study participants were recruited from healthy pregnant women seeking routine gynecological care at the Outpatient Clinic of the Department of Obstetrics and Gynaecology of the University Hospital Würzburg at the beginning of the third trimester of pregnancy (> week 28). They were informed about the aims, risks and benefits of the study and were asked for participation. Those willing to participate were subsequently screened including an intra‐oral examination to verify whether they met the following inclusion criteria:

- age ≥ 21 years
- uneventful pregnancy at the beginning of the third trimester
- signs of gingival inflammation (Gingival Index ≥1 at the buccal aspect of at least one of the Ramfjord teeth 16, 21, 24, 36, 41, 44 (Fleiss et al. 1987).

Exclusion criteria were:

- any preceding adverse event during pregnancy
- manifestation of systemic diseases interfering with gingival inflammation (e.g. diabetes)
- periodontal pockets >5 mm (PSI >3)
- antibiotic therapy within the last 6 months
- use of antibacterial mouth rinses
- intake of anti‐inflammatory drugs > 1×/month
- known allergies towards the ingredients of the experimental lozenges
- inability to comprehend and to comply with the study protocol

### Baseline examination

At baseline the extent of gingival inflammation was assessed visually on the vestibular aspects of the Ramfjord teeth 16, 21, 24, 36, 41, 44 using the Lobene modification of the Gingival Index (Lobene et al. 1986). Subsequently also plaque coverage was assessed on the vestibular surfaces of the Ramfjord teeth using the Plaque Index (Silness & Loe 1964), pooled subgingival bacterial plaque samples were taken from the Ramfjord teeth for the analysis of *P. intermedia* colonization, and 10 ml of a venous blood sample was taken for the determination of TNF‐*α* serum levels.

Using a random list the study participants finally were provided with 8 bottles containing 8 × 28 = 224 experimental lozenges (test/placebo) and instructed to consume them 2× daily until the end of pregnancy. Lozenges not being consumed during the experimental period had to be returned at the end of the study. No specific oral hygiene instructions were given to the study participants other than the encouragement to continue steadily with their usual brushing habits.

### Reevaluation

Within 2 days after birth (approximately 7 weeks after baseline examination) the study participants were reevaluated. Gingival Index and Plaque Index were reassessed and a venous blood sample as well as subginigval plaque samples were taken as described before. Finally compliance with the study protocol was verified by face to face interview and total consumption of the lozenges calculated from the number of returned lozenges.

### Microbial analysis

Pooled bacterial samples were taken from the gingival sulcus of the Ramfjord teeth at baseline and at the end of the study by sterile paper points. They were subsequently analysed for the presence of the pregnancy‐associated periodontopathogen *Prevotella intermedia* by a microbiological real‐time PCR test with a detection limit of ≥100 bacteria per sample.

### TNF‐α serum levels

Analysis of TNF‐*α* serum levels was performed using a commercially available ELISA kit (Quantikine^®^ELISA, Human TNF‐*α* Immunoassay, R&D Systems, Minneapolis, MN, USA).

### Statistical analysis

#### Primary endpoint

The primary endpoint of the study was the assessment of changes in the extent of gingival inflammation as measured by the Lobene modification of the Gingival Index.

#### Group size calculation

With an assumed mean GI of GI = 0.9 at baseline, a standard deviation for GI of ±0.5 and in order to verify a difference in the recorded mean of 50% between test and placebo at reevaluation with a power of 0.9, a sample size of 2 × 26 study patients had been calculated.

As normal distribution for the recorded data could not be assumed, Mann–Whitney *U*‐test was used for the analysis of independent samples, and the Wilcoxon signed rank test for the analysis of paired samples. The level of significance was set to *p* ≤ 0.05. All statistical analyses were performed by a professional statistician using the WinMEDAS statistical software package (pdv software, Goslar, Germany).

### Blinding and randomization

A computer‐generated random list in blocks of six was used for allocating study participants either to the test or the placebo group. Assignment to a participant number was done according to the chronological order of enrolment in the study.

All clinical examinations were performed by a trained and calibrated dentist, who was neither involved in the group assignments nor in the code labelling and the delivery of the experimental lozenges to the study participants. A code break for the randomization was kept in a sealed envelope at the Department of Periodontology.

## Results

### Compliance

**Figure 1:**
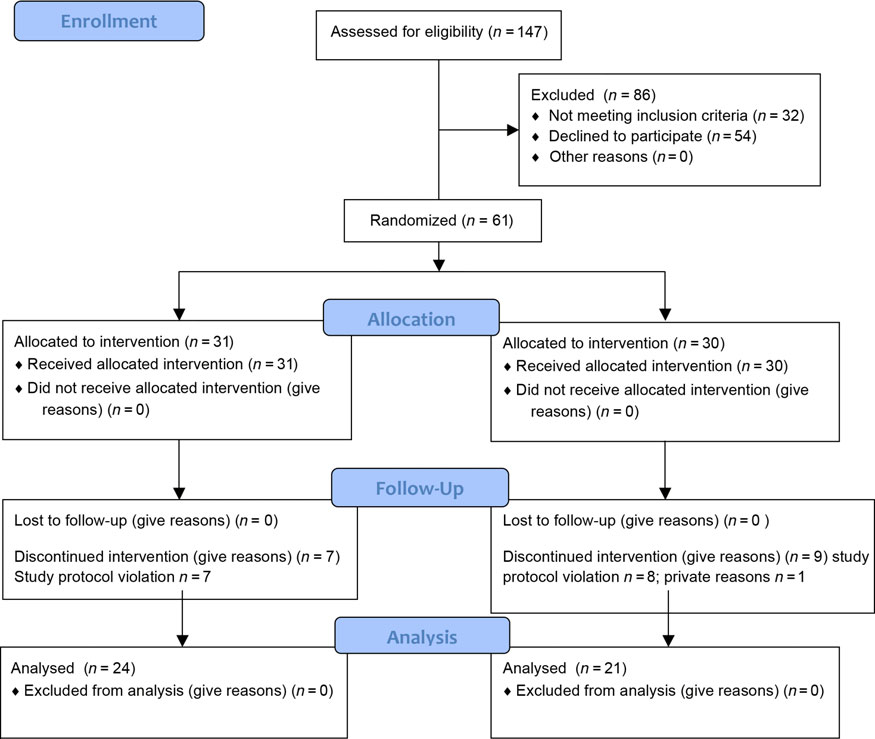
Study design flowchart.

Of a total of 147 screened pregnant women 61 subjects, age 24–40 (mean: 31.4 years ± 3.8 SD) met the inclusion criteria and were scheduled for baseline examination after having given informed consent for study participation. Sixteen of them terminated participation prematurely. Nine had been assigned to the placebo and seven to the test (*L. reuteri*) group. The reasons for leaving the study were the administration of antibiotics or other pregnancy complications not related to the consumption of the study lozenges, but leading to the immediate termination of study participation due to the requirements of the study protocol. Only one study subject left the study prematurely for private reasons. None the recruited participants was in need for dental treatment at baseline or visited a dentist during the course of the study. Forty‐five participants (24 test/21placebo) completed the trial and were reevaluated. Their data were included in the intention to treat analyses presented in the following (Fig. 1).

Due to the reduced number of 24 + 21 recruited study participants test power was recalculated in a post hoc power analysis using the recorded data for the primary endpoint (GI) at the end of the study. It revealed a test power of > 90%, for *p* ≤ 0.05.

### Duration and extent of lozenges consumption (baseline ‐reevaluation)

Due to a possible study participation throughout the complete third trimester of pregnancy and in order to include an additional safety supply for eventually lost lozenges all participants received a supply of 8 × 28 = 224 lozenges sufficient for 112 days of study participation. The actual mean duration of the lonzenge consumption period however was considerably shorter being 41.9 days ±16.0 SD for the test group and 46.3 days ±14.9 SD for the placebo group.

From the number of lozenges brought back by the study subjects’ consumption compliance was calculated. Mean number of lozenges missing, i.e. probably consumed or left at home, was 102.7 ± 31.1 in the test group and 118.2 ± 40.2 in the placebo group, indicative of a consumption of 2,45 lozenges/day in the test group and 2,55 lozenges/day in the placebo group suggesting a good consumption compliance. Differences between the groups were not significant.

### Gingival index (GI), plaque index (PlI), TNF‐α serum levels

The results of the analysis of the recorded GI and PlI scores as well as TNF‐*α* serum levels are shown in Table 1.

**Table 1:** Analysis of Gingival Index (GI) scores, Plaque Index (PlI) scores and TNF‐*α* serum levels

	Test (*L. reuteri*) Group *n* = 24			Placebo Group *n* = 21			*p* a
	Mean	SD	Median	Mean	SD	Median	
GI baseline	1.0	0.6	0.8	0.9	0.6	0.8	n.s.
GI reevaluation	0.2	0.4	0.2	0.7	0.5	0.7	<0.0001
PlI baseline	0.7	0.5	0.7	0.8	0.6	0.7	n.s.
PlI reevaluation	0.2	0.3	0.0	0.6	0.6	0.5	<0.0001
TNF‐*α* serum level baseline (pg/ml)	13.3	1.4	13.4	11.8	1.8	12.2	<0.02
TNF‐*α* serum level reevaluation (pg/ml)	11.7	2.1	12.2	10.7	2.6	11.6	n.s.

a Mann–Whitney *U*‐test.

### Gingival index

**Figure 2:**
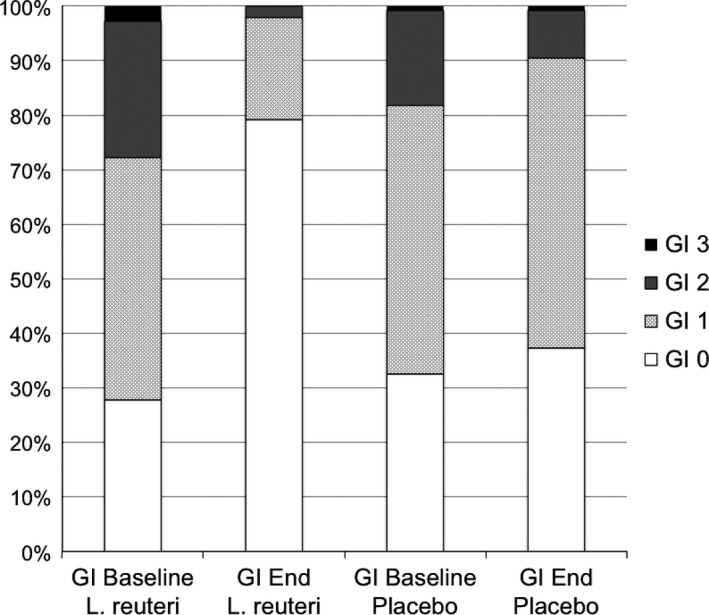
Frequency distribution of the observed Gingival Index (GI) categories GI 1–3.

At baseline there was no significant difference between both experimental groups. At reevaluation, however, the mean GI score of the test group was significantly (*p* < 0.0001) reduced compared to baseline and was significantly (*p* < 0.0001) lower than in the placebo group. Differences between baseline and reevaluation observed for the placebo group failed to reach the level of significance. The frequency distribution of the different GI categories recorded at baseline and at the end of the study is depicted in Fig. 2.

### Plaque index

At baseline there was no significant difference between both groups. At reevaluation by contrast, the recorded mean PlI score of the test group was significantly (*p* < 0.0001) lower compared to baseline and also significantly lower compared to the placebo group (*p* < 0.0001). In the placebo group differences between baseline and reevaluation could not be verified statistically.

### TNF‐α serum levels

At baseline mean TNF‐*α* serum level in the test group was significantly (*p* < 0.02) higher than in the placebo group. At reevaluation by contrast differences between the groups could no longer be verified statistically.

### *P. intermedia* colonization

**Figure 3:**
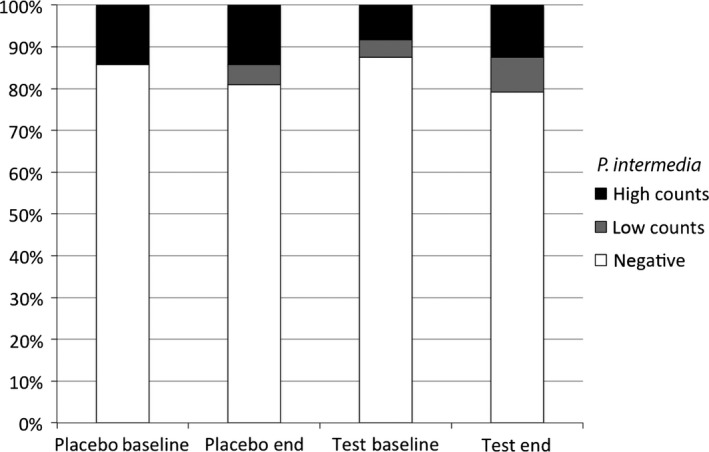
Frequency distribution of *Prevotella intermedia* colonization.

The frequency distribution of detectable *P. intermedia* colonization in the study patients is depicted in Fig. 3. Frequency of *P. intermedia* colonization was low at baseline for both experimental groups (14% *versus* 12%) and was only moderately higher at the end of the study (19% versus 21%). The increase was mostly due to patients being negative at baseline and exhibiting only low *P. intermedia* counts ≤10^3^ bacterial units at the end of the study. Differences between the groups could not be verified statistically neither at baseline nor at the end of the study.

## Discussion

The results of this investigation clearly demonstrate a significant, major association between the regular consumption of *L. reuteri*‐containing lozenges and the reduction in gingival inflammation and plaque coverage in healthy pregnant women. They confirm the clinical findings of Twetman et al. (2009) reporting a significant beneficial influence of *L. reuteri*‐containing chewing gums on the manifestation of bleeding on probing in a cohort of young adults suffering from chronic gingivitis, but are in contrast with the data of Hallstrom et al. (2013), who failed to detect a significant influence of *L. reuteri*‐containing lozenges on Gingival Index and Plaque Index scores in their model of experimental gingivitis in a cohort of young healthy women. Possible explanations for these seemingly contradictory findings may be primarily found in subject and disease selection, as experimental gingivitis is a disease entity which in several etiological aspects may be quite different from established chronic gingivitis (Deinzer et al. 2007) and may particularly not reflect the specific situation of pregnancy gingivitis being the subject of this investigation. Furthermore, Hallstrom et al. exposed their study subjects to repeated intervals without oral hygiene followed by intensive professional tooth cleaning, while in our study care had been taken not to interfere with the habitual mechanical plaque control of the study participants during the duration of the trial. The marked reduction in mean PlI scores observed for the test group at the time of reevaluation, with an only minor, non‐significant reduction in the placebo group may thus not be primarily ascribed to improved hygiene efforts driven by some kind of *Hawthorne* effect. It rather may reflect reduced gingival inflammation, which is generally accompanied by a reduction in de novo plaque formation (Rowshani et al. 2004) due to a decrease in bacterial substrates emanating from the gingival sulcus. The lack of any major effect on the frequency of *P. intermedia* colonization in the *L. reuteri* group likewise supports the preliminary assumption, that anti‐inflammatory modulation of the host response and not direct interference with competing microorganisms may be the major driving force behind the observed reduction in bacterial plaque masses. This however needs to be verified by further investigations. While Twetman et al. reported a significant decrease in proinflammatory cytokine levels in crevicular fluid following the regular consumption of *L. reuteri* food supplements, we were unable to verify a significant effect of *L. reuteri* consumption on TNF‐*α* serum levels. This may be attributed to the fact, that crevicular fluid sampled from an inflamed gingival sulcus will contain higher levels of proinflammatory cytokines than peripheral blood, where the local influx of proinflammatory cytokines will be markedly diluted. Furthermore recent research efforts identified any uneventful pregnancy as a phase of controlled mild maternal systemic inflammation with elevated serum levels of TNF‐*α* and other pro‐inflammatory cytokines (Calleja‐Agius et al. 2012). Thus, a possible decrease in TNF‐*α* serum levels induced by a reduction in gingival inflammation may have been masked by physiological fluctuations of this cytokine triggered by different stages of pregnancy. Finally all mean TNF‐*α* serum level scores recorded in this study were well within the normal range of this cytokine in healthy individuals.

The recorded mean baseline GI scores of 1.0 and 0.9, respectively, suggest that the pregnant women participating in this trial were suffering only from mild pregnancy‐associated gingivitis. However when looking at the frequency distribution of the observed GI categories (Fig. 2) it must be noted, that in both groups also scores of GI 2 and GI 3, indicative of more severe gingival inflammation, were locally present in up to 28% of the sites evaluated at baseline. The pattern of GI scores recorded at the end of the study clearly demonstrate that particularly in the *L. reuteri* group not only sites with mild gingivitis resolved but also the percentage of sites exhibiting GI categories 2 and 3 pronouncedly decreased. It also has to be noted that these changes occurred without any intentional interference with personal oral hygiene or the performance of professional mechanical plaque removal.

## Limitations and Conclusion

The limited number of participants in this trial may impair the validity of its results for drawing general conclusions on the administration of *L. reuteri* food supplements in the therapy of pregnancy associated gingivitis. The health‐related background data of the study participants shown in Table 2 indicate that all of them had an overall status of mostly uncompromised general and oral health with no significant differences between the groups. Thus, it has to be noted that the encouraging findings of this study derived from a cohort of healthy individuals may not allow drawing general conclusions regarding the usefulness of probiotic *L. reuteri* administration in pregnant women with severely compromised general and/or oral health without further evaluations in clinical trials. In the observed cohort of healthy pregnant women however the regular consumption of *L.reuteri*‐containing lozenges proved to be a valuable adjunct in the control of pregnancy‐associated gingivitis being effective even in the intentional absence of concomitant professional plaque removal and oral hygiene training.

**Table 2:** General health, medication and mode of birth profile

	Placebo group (*n* = 21)	Test group (*n* = 24)	*p* a
Age (years)	31 ± 3.3	31.8 ± 4.2	n.s.
Body Height (m)	168 ± 5.7	166 ± 5.78	n.s.
Body Weight (kg)	83.8 ± 18.2	80.1 ± 14.1	n.s.
Non‐smoker	19	23	n.s.
Hypertension	0	1	n.s
Folic acid supplement	9	10	n.s
Birth mode spontaneously	13	17	n.s
Birth mode primary sectio	4	5	n.s
Birth mode secondary sectio	4	1	n.s
Week of gestation at delivery	39.8 ± 1.4	39.7 ± 1.2	n.s.
Duration of lozenge consumption (days)	46.3 ± 14.9	41.9 ± 16.0	n.s.

a *p* Mann–Whitney *U*‐Test, n.s., not significant.

**Clinical Relevance**

*Scientific rationale for the study*: Pregnancy‐associated gingivitis is a frequent accompaniment of gravidity. While the resolution of pregnancy‐associated gingivitis by strict mechanical plaque control is well‐established, the impact of the regular consumption of host response modulating *probiotics* on gingival inflammation in pregnant women has not been evaluated so far.

*Principal findings*: The regular consumption of *L. reuteri*‐containing lozenges during the third trimester of pregnancy was associated with a significant reduction in gingival inflammation and plaque coverage.

*Practical implications*: The regular consumption of *L. reuteri*‐containing lozenges may have the potential to become an effective adjunct in the control of pregnancy‐associated gingivitis.

## References

[jcpe12606-bib-0001] Alexander, K. L. , Targan, S. R. & Elson, C. O. 3rd (2014) Microbiota activation and regulation of innate and adaptive immunity. Immunological Reviews 260, 206–220. 24942691 10.1111/imr.12180 PMC4080089

[jcpe12606-bib-0002] Baca‐Castanon, M. L. , De la Garza‐Ramos, M. A. , Alcazar‐Pizana, A. G. , Grondin, Y. , Coronado‐Mendoza, A. , Sanchez‐Najera, R. I. , Cardenas‐Estrada, E. , Medina‐De la Garza, C. E. & Escamilla‐Garcia, E. (2015) Antimicrobial Effect of *Lactobacillus reuteri* on Cariogenic Bacteria *Streptococcus gordonii, Streptococcus mutans*, and Periodontal Diseases *Actinomyces naeslundii* and *Tannerella forsythia* . Probiotics Antimicrob Proteins 7, 1–8. 25422124 10.1007/s12602-014-9178-y

[jcpe12606-bib-0003] Calleja‐Agius, J. , Muttukrishna, S. & Jauniaux, E. (2012) IThe role of tumor necrosis factor‐receptors in pregnancy with normal and adverse outcome. International Journal of Interferon, Cytokine and Mediator Research 4, 1–15.

[jcpe12606-bib-0004] Deinzer, R. , Weik, U. , Kolb‐Bachofen, V. & Herforth, A. (2007) Comparison of experimental gingivitis with persistent gingivitis: differences in clinical parameters and cytokine concentrations. Journal of Periodontal Research 42, 318–324. 17559628 10.1111/j.1600-0765.2006.00951.x

[jcpe12606-bib-0005] Fleiss, J. L. , Park, M. H. , Chilton, N. W. , Alman, J. E. , Feldman, R. S. & Chauncey, H. H. (1987) Representativeness of the “Ramfjord teeth” for epidemiologic studies of gingivitis and periodontitis. Community Dentistry and Oral Epidemiology 15, 221–224. 3476248 10.1111/j.1600-0528.1987.tb00525.x

[jcpe12606-bib-0006] Forsberg, A. , Abrahamsson, T. R. , Bjorksten, B. & Jenmalm, M. C. (2013) Pre‐ and post‐natal *Lactobacillus reuteri* supplementation decreases allergen responsiveness in infancy. Clinical and Experimental Allergy 43, 434–442. 23517039 10.1111/cea.12082

[jcpe12606-bib-0007] Fteita, D. , Kononen, E. , Gursoy, M. , Soderling, E. & Gursoy, U. K. (2015) Does estradiol have an impact on the dipeptidyl peptidase IV enzyme activity of the *Prevotella intermedia* group bacteria? Anaerobe 36, 14–18. 26386229 10.1016/j.anaerobe.2015.09.002

[jcpe12606-bib-0008] Ganzle, M. G. , Holtzel, A. , Walter, J. , Jung, G. & Hammes, W. P. (2000) Characterization of reutericyclin produced by *Lactobacillus reuteri* LTH2584. Applied and Environment Microbiology 66, 4325–4333. 10.1128/aem.66.10.4325-4333.2000 PMC92303 11010877

[jcpe12606-bib-0009] Hallstrom, H. , Lindgren, S. , Yucel‐Lindberg, T. , Dahlen, G. , Renvert, S. & Twetman, S. (2013) Effect of probiotic lozenges on inflammatory reactions and oral biofilm during experimental gingivitis. Acta Odontologica Scandinavica 71, 828–833. 23294143 10.3109/00016357.2012.734406

[jcpe12606-bib-0010] Iniesta, M. , Herrera, D. , Montero, E. , Zurbriggen, M. , Matos, A. R. , Marin, M. J. , Sanchez‐Beltran, M. C. , Llama‐Palacio, A. & Sanz, M. (2012) Probiotic effects of orally administered *Lactobacillus reuteri*‐containing tablets on the subgingival and salivary microbiota in patients with gingivitis. A randomized clinical trial. Journal of Clinical Periodontology 39, 736–744. 22694350 10.1111/j.1600-051X.2012.01914.x

[jcpe12606-bib-0011] Kaur, M. , Geisinger, M. L. , Geurs, N. C. , Griffin, R. , Vassilopoulos, P. J. , Vermeulen, L. , Haigh, S. & Reddy, M. S. (2014) Effect of intensive oral hygiene regimen during pregnancy on periodontal health, cytokine levels, and pregnancy outcomes: a pilot study. Journal of Periodontology 85, 1684–1692. 25079400 10.1902/jop.2014.140248 PMC4372244

[jcpe12606-bib-0012] Kornman, K. S. & Loesche, W. J. (1980) The subgingival microbial flora during pregnancy. Journal of Periodontal Research 15, 111–122. 6103927 10.1111/j.1600-0765.1980.tb00265.x

[jcpe12606-bib-0013] Kumar, P. S. (2013) Sex and the subgingival microbiome: do female sex steroids affect periodontal bacteria?. Periodontology 2000 61, 103–124. 23240946 10.1111/j.1600-0757.2011.00398.x

[jcpe12606-bib-0014] Lima, D. P. , Moimaz, S. A. , Garbin, C. A. , Sumida, D. H. , Jardim, E. G. Jr & Okamoto, A. C. (2015) Occurrence of socransky red complex in pregnant women with and without periodontal disease. Oral Health & Preventive Dentistry 13, 169–176. 25386627 10.3290/j.ohpd.a32989

[jcpe12606-bib-0015] Lobene, R. R. , Weatherford, T. , Ross, N. M. , Lamm, R. A. & Menaker, L. (1986) A modified gingival index for use in clinical trials. Clinical Preventive Dentistry 8, 3–6. 3485495

[jcpe12606-bib-0016] Loe, H. & Silness, J. (1963) Periodontal Disease in Pregnancy. I. Prevalence and Severity. Acta Odontologica Scandinavica 21, 533–551. 14121956 10.3109/00016356309011240

[jcpe12606-bib-0017] Mazmanian, S. K. , Round, J. L. & Kasper, D. L. (2008) A microbial symbiosis factor prevents intestinal inflammatory disease. Nature 453, 620–625. 18509436 10.1038/nature07008

[jcpe12606-bib-0018] Niederman, R. (2013) Pregnancy gingivitis and causal inference. Evidence‐Based Dentistry 14, 107–108. 24357820 10.1038/sj.ebd.6400966

[jcpe12606-bib-0019] Poutahidis, T. , Kearney, S. M. , Levkovich, T. , Qi, P. , Varian, B. J. , Lakritz, J. R. , Ibrahim, Y. M. , Chatzigiagkos, A. , Alm, E. J. & Erdman, S. E. (2013) Microbial symbionts accelerate wound healing via the neuropeptide hormone oxytocin. PLoS One 8, e78898. 24205344 10.1371/journal.pone.0078898 PMC3813596

[jcpe12606-bib-0020] Raber‐Durlacher, J. E. , Leene, W. , Palmer‐Bouva, C. C. , Raber, J. & Abraham‐Inpijn, L. (1993) Experimental gingivitis during pregnancy and post‐partum: immunohistochemical aspects. Journal of Periodontology 64, 211–218. 8463944 10.1902/jop.1993.64.3.211

[jcpe12606-bib-0021] Raber‐Durlacher, J. E. , van Steenbergen, T. J. , Van der Velden, U. , de Graaff, J. & Abraham‐Inpijn, L. (1994) Experimental gingivitis during pregnancy and post‐partum: clinical, endocrinological, and microbiological aspects. Journal of Clinical Periodontology 21, 549–558. 7989619 10.1111/j.1600-051x.1994.tb01172.x

[jcpe12606-bib-0022] Rowshani, B. , Timmerman, M. F. & Van der Velden, U. (2004) Plaque development in relation to the periodontal condition and bacterial load of the saliva. Journal of Clinical Periodontology 31, 214–218. 15016026 10.1111/j.0303-6979.2004.00468.x

[jcpe12606-bib-0023] Sansonetti, P. J. (2011) To be or not to be a pathogen: that is the mucosally relevant question. Mucosal Immunology 4, 8–14. 21150896 10.1038/mi.2010.77

[jcpe12606-bib-0024] Silness, J. & Loe, H. (1964) Periodontal disease in pregnancy. II. Correlation between oral hygiene and periodontal condtion. Acta Odontologica Scandinavica 22, 121–135. 14158464 10.3109/00016356408993968

[jcpe12606-bib-0025] Simon, M. C. , Strassburger, K. , Nowotny, B. , Kolb, H. , Nowotny, P. , Burkart, V. , Zivehe, F. , Hwang, J. H. , Stehle, P. , Pacini, G. , Hartmann, B. , Holst, J. J. , MacKenzie, C. , Bindels, L. B. , Martinez, I. , Walter, J. , Henrich, B. , Schloot, N. C. & Roden, M. (2015) Intake of *Lactobacillus reuteri* improves incretin and insulin secretion in glucose‐tolerant humans: a proof of concept. Diabetes Care 38, 1827–1834. 26084343 10.2337/dc14-2690

[jcpe12606-bib-0026] Stensson, M. , Koch, G. , Coric, S. , Abrahamsson, T. R. , Jenmalm, M. C. , Birkhed, D. & Wendt, L. K. (2014) Oral administration of *Lactobacillus reuteri* during the first year of life reduces caries prevalence in the primary dentition at 9 years of age. Caries Research 48, 111–117. 24296746 10.1159/000354412

[jcpe12606-bib-0027] Talarico, T. L. , Casas, I. A. , Chung, T. C. & Dobrogosz, W. J. (1988) Production and isolation of reuterin, a growth inhibitor produced by *Lactobacillus reuteri* . Antimicrobial Agents and Chemotherapy 32, 1854–1858. 3245697 10.1128/aac.32.12.1854 PMC176032

[jcpe12606-bib-0028] Tekce, M. , Ince, G. , Gursoy, H. , Dirikan Ipci, S. , Cakar, G. , Kadir, T. & Yilmaz, S. (2015) Clinical and microbiological effects of probiotic lozenges in the treatment of chronic periodontitis: a 1‐year follow‐up study. Journal of Clinical Periodontology 42, 363–372. 25728888 10.1111/jcpe.12387

[jcpe12606-bib-0029] Teughels, W. , Durukan, A. , Ozcelik, O. , Pauwels, M. , Quirynen, M. & Haytac, M. C. (2013) Clinical and microbiological effects of *Lactobacillus reuteri* probiotics in the treatment of chronic periodontitis: a randomized placebo‐controlled study. Journal of Clinical Periodontology 40, 1025–1035. 24164569 10.1111/jcpe.12155 PMC3908359

[jcpe12606-bib-0030] Twetman, S. , Derawi, B. , Keller, M. , Ekstrand, K. , Yucel‐Lindberg, T. & Stecksen‐Blicks, C. (2009) Short‐term effect of chewing gums containing probiotic *Lactobacillus reuteri* on the levels of inflammatory mediators in gingival crevicular fluid. Acta Odontologica Scandinavica 67, 19–24. 18985460 10.1080/00016350802516170

[jcpe12606-bib-0031] Wu, M. , Chen, S. W. & Jiang, S. Y. (2015) Relationship between gingival inflammation and pregnancy. Mediators of Inflammation 2015, 623427. 25873767 10.1155/2015/623427 PMC4385665

